# *DNMT3A* Deficiency Reduces *DNMT3B* Gene Methylation and Contributes to Whole-genome Transcription Alterations in HEK293 Cells

**DOI:** 10.2174/0113892029351729250217113313

**Published:** 2025-02-24

**Authors:** Mengxiao Zhang, Jiaxian Wang, Gen Qi, Lanfeng Xie, Qiuxiang Tian, Hui Yang, Lei Feng, Nan Zhu, Xingchen Pan, Jianwei Zhu, Jianjun Hu, Peng Chen, Huili Lu

**Affiliations:** 1 Engineering Research Center of Cell and Therapeutic Antibody, Ministry of Education, School of Pharmacy, Shanghai Jiao Tong University, Shanghai, China;; 2 Department of Hematology, VU University Medical Center, Amsterdam, Netherlands;; 3 Department of Infectious Disease, Tongren Hospital, School of Medicine, Shanghai Jiao Tong University, Shanghai, China;; 4 Key Laboratory of Pathobiology, Ministry of Education, Jilin University, Changchun, Jilin, China;; 5 Department of Genetics, College of Basic Medical Sciences, Jilin University, Changchun, Jilin, China;; 6 Instrumental Analysis Center, Shanghai Jiao Tong University, Shanghai, China;; 7 Shanghai General Hospital, School of Medicine, Shanghai Jiao Tong University, Shanghai, China

**Keywords:** DNMT3A, HEK293, CRISPR/Cas9, RNA-seq, methylation, DNA

## Abstract

**Introduction:**

DNA methylation is an important epigenetic modification associated with transcriptional repression and plays key roles in normal cell growth as well as oncogenesis. Among the three main DNA methyltransferases (DNMT1, DNMT3A, and DNMT3B), DNMT3A mediates *de novo* DNA methylation. However, the general effect of DNMT3A on cell proliferation, metabolism, and downstream gene regulation is still to be unveiled.

**Methods:**

In this study, we successfully created *DNMT3A*-deficient HEK293 cells with frameshift mutations in the catalytic domain using CRISPR/Cas9 technology. The *DNMT3A* deficient cells showed a 21.5% reduction in global DNA methylation levels, leading to impaired cell proliferation as well as a blockage of MAPK and PI3K-Akt pathways in comparison with wild-type cells.

**Results and Discussion:**

RNA-seq analysis demonstrated that *DNMT3A* knockout resulted in the up-regulation of genes and pathways related to cell metabolism but down-regulation of those involved in ribosome function, potentially explaining the growth and signaling pathways inhibition. Furthermore, DNMT3A ablation reduced *DNMT3B* gene methylation, explaining the down-regulated profiles of genes.

**Conclusion:**

Our findings suggest a complex epigenetic regulatory role for *DNMT3A*, and the compensatory upregulation of *DNMT3B* in response to *DNMT3A* deficiency warrants further investigation to be validated in future studies.

## 1 INTRODUCTION

DNA methylation is an epigenetic modification with widespread effects on gene expression. High levels of promoter DNA methylation are usually associated with gene silencing [1]. Abnormal DNA methylation is involved in the development of multiple malignancies, such as solid tumors and leukemia [2-4]. In vertebrates, cytosine methylation in CpG dinucleotides is the predominant form of methylation catalyzed by DNA methyltransferase 1 (DNMT1) [5, 6] and established *de novo* by DNMT3A and DNMT3B [7, 8].

To investigate the mechanisms responsible for locus-specific and global methylation, *in vivo* and *in vitro* models of DNMT deficiency have been developed [9]. In mice, knockout of *DNMT1* or *DNMT3B* can cause early embryo death. In contrast, *DNMT3A* knockout mice can be born normally but develop developmental defects and die prematurely soon after birth [7]. These observations indicate that DNMT3A plays specific roles in regulating chromatin methylation during development after birth [7, 10]. Similarly, in human embryonic cells, individual or simultaneous disruption of DNMT3A and DNMT3B resulted in viable, pluripotent cell lines, but deletion of DNMT1 resulted in rapid cell death [11]. Banaszak *et al.* mutated DNMT3A in K562 leukemia cells, and the derived cell lines showed impaired cell growth [2]. Although almost all cells can survive DNMT3A mutation, reports have demonstrated paradoxical hyper-methylation of genes downstream of DNMT3A or no changes in global or regional DNA methylation patterns in response to DNMT3A knockdown [2, 12]. Hence, the exact roles of DNMT3A are yet to be elucidated.

The CRISPR/Cas9 system is an efficient genome editing technique that was developed in recent years [13, 14]. Compared with the traditional knockout techniques, such as zinc finger nuclease technology (ZFN) and transcription activator-like effector nucleases (TALEN), CRISPR/Cas9 is comparatively easy to implement, cost and time-effective, and has a higher efficiency. The CRISPR/Cas9 technique has been successfully used in human cells, zebrafish, mice, and bacterial genome modification [15, 16]. In the present study, we employed the CRISPR/Cas9 technology to establish a *DNMT3A* knockout cell line derived from HEK293T, a human embryonic kidney cell line. We performed detailed transcriptomic and epigenetic analyses, in addition to physiological measurements, to discover the impact of DNMT3A deficiency on cell proliferation and metabolism as well as to identify genes that are potentially regulated by DNMT3A.

## 2 MATERIALS AND METHODS

### 2.1 Cell Culture and Reagents

Wild-type HEK293T cells were obtained from the Type Culture Collection of the Chinese Academy of Sciences (Shanghai, China) and detected to be negative for mycoplasma contamination using the Myco-Blue mycoplasma detector (Vazyme; Nanjing, Jiangsu, China). Cells were cultured in high glucose DMEM supplemented with 10% FBS, incubated at 37°C with 5% CO_2_ in a humidified cell incubator (Thermo Fisher Scientific; Marietta, OH, USA). The plasmid pX330 carrying CRISPR/Cas9 system was kindly provided by Dr. Feng Zhang (MIT) [15]. Competent cells of the *E. coli* strain DH5α were purchased from Microgene (Shanghai, China). All media and supplements were purchased from Gibco (Thermo Fisher Scientific; Waltham, MA, USA). Cell growth and viability were monitored with a cell counter (Countstar; Shanghai, China).

### 2.2 SgRNA Design and DNMT3A Disruptive Vector Construction

Two sgRNAs targeting exon 19 of *DNMT3A* (GeneBank ID 806904736) were designed using the web tool provided by Dr. Zhang’s lab (http://crispr.mit.edu), as shown in Fig. (**1**). To construct the sgRNA plasmids, single strand primers were designed and synthesized as sgRNA1-forward: 5’-CACCGCATGATGCGCGGCCCAAGG-3’, sgRNA1-reverse 5’-AAACCCTTGGGCCGCGCATCATGC-3’, sgRNA2-forward 5’-CACCGCTCACTAATGGCTTCTACCT-3’ and sgRNA1-reverse 5’-AAACAGGTAGAAGCCATTAGTGAGC-3’. Each pair of primers was annealed to generate double-stranded DNA, phosphorylated by T4 polynucleotide kinase at the 5’ ends (NEB, Ipswich, MA) at 37°C for 30 min, and further ligated into BbsI digested pX330 plasmids by T4 DNA ligase (Takara; Kusatsu, Shiga, Japan). The ligate was transformed to DH5α competent cells for culture overnight. Then, the grown clones were selected for sequencing to get the correct constructed plasmids pX330-sgRNA1 and pX330-sgRNA2.

### 2.3 Transfection of HEK293T Cells

HEK293T cells were seeded at 2×10^5^ cells/well into a 12-well plate one day prior to transfection. When reached 70-80% confluence, the cells were co-transfected with pX330-sgRNA1 and pX330-sgRNA2 at a molar ratio of 1:1, as reported that double sgRNAs could result in a higher editing efficiency than single one [17]. The transfection was performed using Lipofectamine 2000 reagent (Invitrogen; Carlsbad, CA, USA) according to the manufacturer’s instructions.

### 2.4 DNMT3A Knockout Clones Selection

HEK293T cell pool transfected with pX330-sgRNAs was seeded into 96-well plates at the density of 0.5 cells per well for limiting dilution. After about ten days of incubation, the plates were examined for single-cell clones under a microscope. When they grew to about 80% confluent in the well, the clones were detached for subpopulation, and the genomic DNA was extracted with *QuickExtract* DNA extraction solution (Epicenter; Edgewood, MD, USA) for PCR verification, using primers HEK293-DNMT3A-For (5’-GTACCATCCTGTCCCCTCCAC-3’) and HEK293-DNMT3A- Rev (5’-GGCTCAGGGTTAAACGGGGA-3’), which could amplify a 798 bp fragment for HEK293T wild-type cells. By sequencing the amplified fragments, the clone with disrupted DNMT3A was selected and designated to be the DNMT3A KO cell line.

### 2.5 DNMT3A Knockout Cells Proliferation Curve

DNMT3A KO and wild-type (WT) cells were seeded at 3×10^4^ cells/well into a 12-well plate. Cells were counted every 24 h for the consecutive 6 days. Cell proliferation curves were compared between the two cell lines.

### 2.6 Western Blot Analysis

For the western blot analysis, *1 ×*106 cells of DNMT3A KO and WT HEK293T were washed with PBS, lysed using 100 μL RAPA lysis buffer containing protease inhibitors cocktail (Roche; Penzberg, Germany), and separated by a 10% SDS-PAGE. After transferring onto a 0.45 μm PVDF membranes, immunoblotting was performed to detect the expression of DNMT3A and primary mouse monoclonal antibody against GAPDH (Sangon; Shanghai, China) and polyclonal rabbit-anti-human DNMT3A (Sangon) were used at 1:1000 dilution. For the detection of MAPK and PI3K-Akt pathways, primary monoclonal antibodies against human Erk (137F5; Cell Signaling Technology; Danvers, MA, USA), phosphor-Erk (197G2; Cell Signaling Technology), JNK (D-2; Santa Cruz; Dallas, TX, USA), phosphor-JNK (G9; Cell Signaling Technology), Akt (11E7; Cell Signaling Technology), and phosphor-Akt (244F9; Cell Signaling Technology) were used. HRP-conjugated anti-mouse IgG or anti-rabbit IgG antibodies (Jackson ImmunoResearch; West Grove, PA, USA) were used as secondary antibodies. Signals were detected with enhanced chemiluminescence (Millipore; Billerica, MA, USA) and visualized with a gel imaging system (Tanon; Shanghai, China).

### 2.7 Genome-wide DNA Methylation Analysis by UPLC-ESI-MS/MS

The genomic DNA of cells was extracted by AxyPrep Kit (Axygen; Hangzhou, Zhejiang, China) with the treatment of RNase A for RNA removal. Then, the genomic DNA was hydrolyzed by DNase I at 37°C for 1 h, denatured at 100°C for 3 min, and immediately cooled down on ice for 10 min, subsequently treated with *Nuclease* P1 at 37°C for 16 h, followed by treatment of alkaline phosphatase at 37°C for 2 h. The nucleotides were stored at -20°C before UPLC-ESI-MS-MS detection.

Acquity UPLC (Waters; Milford, MA, USA) coupled with Triple Quad™ 5500 mass spectrometry (Sciex, Framingham, MA, USA) was used to quantitatively analyze m^5^dC and dG. UPLC-ESI-MS/MS method was established to evaluate the DNA methylation status of the genome [18]. Reference nucleotide standards of A, G, T, C, dA, dG, dC, U, and m^5^dC were purchased from Sigma (Sigma Aldrich; St. Louis, MO, USA) and dissolved in H_2_O to a final concentration of 1.0 mg/ml. UPLC and electronic spray were used to separate and detect the standards at multiple reaction monitoring modes. The m^5^dC(m/z 241.9→126.3)and dG (m/z 268.1→152.3) were chosen as parent and child ion pairs for quantitative detection. The CE voltage of both m^5^dC and dG was 15 eV, and the DP voltage was 40 V, respectively. Standard curves of m^5^dC and dG were first graphed, and the level of cytosine methylation was calculated as (m^5^dC/dG) x 100% as the content of dC equals to that of dG.

### 2.8 RNA-seq to Reveal Transcriptional Response to DNMT3A Deficiency

Total RNA was extracted from 10^6^ cells of DNMT3A KO or WT, followed by mRNA enrichment with OligodT magnetic beads. CDNA was obtained using Illumina Truseq^TM^ RNA sample prep Kit, and pair-end sequencing (insert size = 300 bp, read length = 150 bp) was performed according to the standard protocol of Novaseq 6000 (Illumina; San Diego, CA, USA). Raw sequencing reads were filtered to include only high-quality reads in downstream analysis: 1) clip adapter sequence from reads, and remove reads with no insertion; 2) clip 3’ low-quality bases (Phred quality < 20), and remove the whole read if there exists a single base with Phred quality < 10; 3) remove the reads that have more than 10% ambiguous bases (N); and 4) remove the reads that are shorter than 20 bp after clipping. The filtered reads were aligned to the human transcriptome (build GRCh38) by TopHat [19]. PCR duplicates were marked and ignored in downstream analysis. All the data were deposited into the open-access Genome Sequence Archive (gsa.big.ac.cn) under accession no. CRA002294.

The read count data of DNMT3A KO and WT cells was analyzed by Cufflink software to identify the differential gene expression induced by DNMT3A deficiency [20]. We used FPKM (fragments per kilobase of exon model per million mapped reads) to estimate gene expression levels. False discovery rate (FDR) *p*-values were calculated using the method proposed by Benjamini and Hochberg (1995) to correct for multiple testing. Differentially expressed genes in DNMT3A KO cells were identified by FDR *p*-value ≤ 0.05 and the absolute logarithm of fold change (log_2_FC) ≥ 2.

### 2.9 KEGG Pathway Analysis of Differentially Expressed Genes

For the purpose of pathway enrichment analysis, we defined differential expression (FDR *p*-value ≤ 0.05 and absolute log_2_FC ≥ 1). The Ensembl IDs of differentially expressed genes were analyzed by KOBAS (http://kobas.cbi.pku.edu.cn) for KEGG pathway enrichment. The pathways with FDR *p*-value ≤ 0.05 were considered significantly differentially expressed.

### 2.10 Bisulfite DNA Analysis and Quantitative PCR Verification of DNMT3A-regulated Genes

DNMT3A is responsible for the *de novo* methylation of multiple genes, and its mutation can lead to demethylation of promoter CpG and thus elevate gene expression at the transcript level, which will further regulate downstream genes indirectly. To delineate the potential regulations of some downstream genes by DNMT3A from the gene pool in which transcript level was interfered by DNMT3A knockout as determined by RNA-seq, we selected three representative genes to be validated by bisulfite DNA analysis as well as quantitative PCR: RUNX1, IQGAP3, and DNMT3B. RUNX1 is known to be regulated by DNMT3A in hematopoietic carcinogenesis [21]. IQGAP3 is a scaffolding protein that is involved in cancer cell proliferation and with no correlation with DNA methyltransferases reported before [22]. All three genes were studied in malignancy development and are helpful in understanding the functions of DNMT3A.

DNA methylation status of selected genes was analyzed by bisulfite sequencing PCR (BSP). Genomic DNA was extracted with an Axygen Genomic DNA Miniprep Kit (San Francisco, CA, USA), and 0.5 μg of DNA was modified through bisulfite treatment using a Bisuldream^®^ — Methylation Universal kit (Miozyme; Shanghai, China). Bisulfite-PCR of the genes promoter regions (Table **S1**) was performed using the following specific primers: RUNX1 forward: 5’- TTTTTAGGTTTTAAAATATTTGTGAGTTGT-3’, RUNX1 reverse: 5’- CACCTACCCTCCCCCAAACTATAC-3’, IAGAP3 forward: 5’- GTAGAAAAGGAGTTTGGAAGGAATAAGA-3’, IQGAP3 reverse: 5’- ACTCACAAACTACCCAACCTAAACC-3’, and DNMT 3B forward 5’- TTAAAGTAGGATGATAGGTAGGGGTAT-3’, DNMT3B reverse: 5’- CCCTAAAAAATCAAAAACCCTAAAC-3’. The amplified fragments were inserted into pMD19-T vectors (Takara; Tokyo, Japan), and 10-15 clones for each gene were selected for sequencing. The results were analyzed by a web-based quantification tool for methylation analysis (http://quma.cdb.riken.jp).

To detect the transcription levels of the above selected three genes, the DNMT3A KO and WT HEK293T cells were cultured, and RNA samples were extracted using a Direct-zol RNA kit (Zymo Research; Irvine, CA, USA). Then, cDNA was synthesized according to the protocol of the RT-PCR kit (Takara; Kusatsu, Shiga, Japan) and used as templates for quantitative PCR. The primers were designed using Primer Premier 5.0 (Premier Biosoft; Palo Alto, CA, USA) according to published sequences (NCBI Accession number: D43967 for RUNX1, AB105103 for IQGAP3, AF156487 for DNMT3B, and M33197 for GAPDH). The following sequences for primers were synthesized (Sangon Biotech; Shanghai, China) as RUNX1 forward: 5' – TCTCTTCCTCTATCTTCCA– 3’, RUNX1 reverse: 5'–GGTATGTGCTATCTGCTTA–3’; IQGAP3 forward: 5'–GACCACTACCTAACTCAG–3’, IQGAP3 reverse 5'–GCATCATCAACAACTTCTA–3’; DNMT3B forward: 5’- GGCAAGTTCTCCGAGGTCTCTG-3’, DNMT3B reverse: 5’-TGGTACATGGCTTTTCGATAGGA-3’; and GAPDH forward: 5'–CTCTGGTAAAGTGGATATTGT–3’, GAPDH reverse: 5'– GGTGGAATCATATTGGAACA–3’). The real-time PCR procedures were performed with 25 μL PCR reaction systems, including 12.5 μL qPCR Mix (Toyobo; Osaka, Japan), 0.4 μM of each primer, and 1 μL template cDNA by thermocycler (StepOnePlus; Foster City, CA, USA). The delta-delta threshold cycle (C_T_) method was used to calculate the expression levels of targeted genes related to the housekeeping gene *GAPDH*.

## 3 RESULTS

### 3.1 Generation of DNMT3A-deficient Clones of HEK293T

**Fig. (2):**
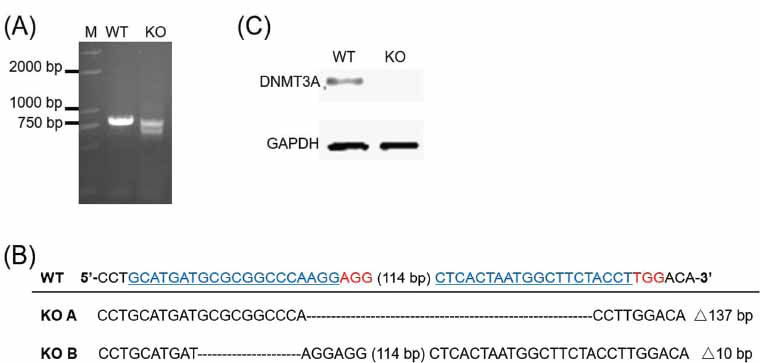
Verification of DNMT3A knockout clone. (**A**) PCR identification of the DNMT3A knockout. Lane M: Trans 2K DNA ladder. (**B**) Sanger sequencing results of PCR amplicons. KO A and KO B represent the two alleles of the *DNMT3A* gene in the DNMT3A KO cells. Blue bases: sgRNA sequences; Red bases: PAM. (**C**) Detection of DNMT3A protein expression through western blotting. WT: wild-type control, KO: DNMT3A KO cells.

The pX330-sgRNA1 and pX330-sgRNA2 plasmids were co-transfected into HEK293T cells. After limiting dilution, clones were selected by PCR using the HEK293-DNMT3A-For and HEK293-DNMT3A-Rev verification primers. We identified one DNMT3A-deficient clone from 17 clones that showed complete disruption of *DNMT3A* and designated it as DNMT3A KO (Fig. **2A**). Fig. (**2B**) shows 137 bp and 10 bp deletions in the KO A and KO B alleles, respectively, leading to the complete ablation of *DNMT3A* due to frameshift mutations. Next, we performed western blotting to characterize the expression of DNMT3A in the DNMT3A KO clone. As shown in Fig. (**2C**), DNMT3A protein expression was completely abrogated in the selected clone, confirming the successful ablation of DNMT3A.

### 3.2 DNMT3A Deficiency Resulted in a Genome-wide Decrease in DNA Methylation

DNMT3A is responsible for the DNA methylation of a large number of genes in mammalian cells. To further verify the effect of *DNMT3A*, we performed UPLC-MS to quantify the global DNA methylation level changes following *DNMT3A* knockout. As described in materials and methods, we first characterized the peaks of the standards A, G, T, C, dA, dG, dC, U, and m^5^dC and then developed the linear curve for dG and m^5^dC (Fig. **S1**). Genomic DNA was extracted from DNMT3A KO and WT cells and hydrolyzed to nucleotides for the measurement of dG and m^5^dC content. The percentage of m^5^dC/dG was calculated to represent the genomic DNA methylation level. As shown in Fig. (**3**), the whole-genome DNA methylation level decreased significantly (by 21.5%) in *DNMT3A* KO cells (12.35 ± 0.36%) compared to that in WT cells (9.69 ± 0.13%).

### 3.3 DNMT3A Deficiency Impaired Cell Growth

To evaluate the effect of DNMT3A deficiency on cell proliferation, the growth profiles of *DNMT3A* KO and WT cells were evaluated. As shown in Fig. (**4**)., the proliferation ability of the HEK293T cells was significantly reduced in response to *DNMT3A* deficiency. After 6 days, the cell count of *DNMT3A* KO cells was reduced to only 40% that of WT cells (0.77 ± 0.15) × 10^6^ *vs* (1.94 ± 0.17) × 10^6^ cells). Further, the doubling time was notably prolonged from 0.99 ± 0.28 days (for WT cells) to 1.53 ± 0.39 days for *DNMT3A* KO cells.

### 3.4 RNA-seq Analysis

After clipping and filtering, RNA-seq yielded sequencing data of 53.2 million reads (7.9 billion base pairs) and 54.8 million reads (8.2 billion base pairs) for *DNMT3A* KO and WT cells, respectively. These data provided 264.8 and 273.5 times coverage of the human transcriptome (30 million base pairs in size). At least 98.4% of the bases had a Phred quality > 20 (error rate < 0.01%). TopHat mapped 94.0% of the sequencing reads to the human genome, with 3.4% of reads mapped to multiple genomic positions that were excluded from the expression analysis.

### 3.5 Differentially Expressed Genes and Pathways

**Fig. (5):**
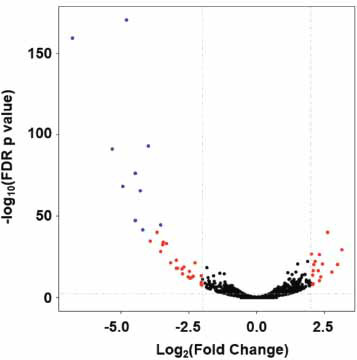
The significance and fold change of differential gene expression induced by *DNMT3A* deficiency. Each dot represents a gene; the significantly differentially expressed genes (FDR *p*-value ≤ 0.05 and fold change ≥ 2) are shown in red; the blue dots indicate the top ten differentially expressed genes (FDR *p*-value ≤2.46×10^-42^).

At a significant FDR *p*-value (≤ 0.05) and absolute log_2_FC ≥ 2, we identified 51 differentially expressed genes (Fig. **5**). Among them, more genes were down-regulated (N = 34) than up-regulated (N = 17). The top ten differentially methylated genes are listed in Table **1** (FDR *p*-value ≤ 2.46×10^-42^). Pathway enrichment analysis was performed for 815 up-regulated and 658 down-regulated Ensembl IDs (FDR *p*-value ≤ 0.01 and absolute fold change ≥ 1.5). Pathways related to calcium signaling, ECM-receptor interaction, and Hippo signaling were up-regulated (Table **2**), while pathways, including those for ribosome biogenesis and cysteine and methionine metabolism, were down-regulated (FDR *p*-value ≤ 0.01, Table **3**).

### 3.6 Methylation Statuses and Transcript Levels of Representative Genes Regulated by DNMT3A Deficiency

RNA-seq showed that many genes were up- and down-regulated upon DNMT3A knockout (Fig. **5**), indicating that DNMT3A deficiency alters gene methylation profiles. Since DNMT3A and DNMT3B are responsible for *de novo* DNA methylation, the RNA-seq signal of DNMT3B was determined. We observed a 1.31-fold increase in the RNA-seq signal of DNMT3B in *DNMT3A* KO cells compared to that in WT cells, indicating the possible compensatory effect of DNMT3B on the deficiency of DNMT3A. Up-regulation of DNMT3B may result in the methylation of some genes and the reduction of their transcription, which may explain why the transcription levels of some genes were reduced after DNM3TA knockout.

**Fig. (6):**
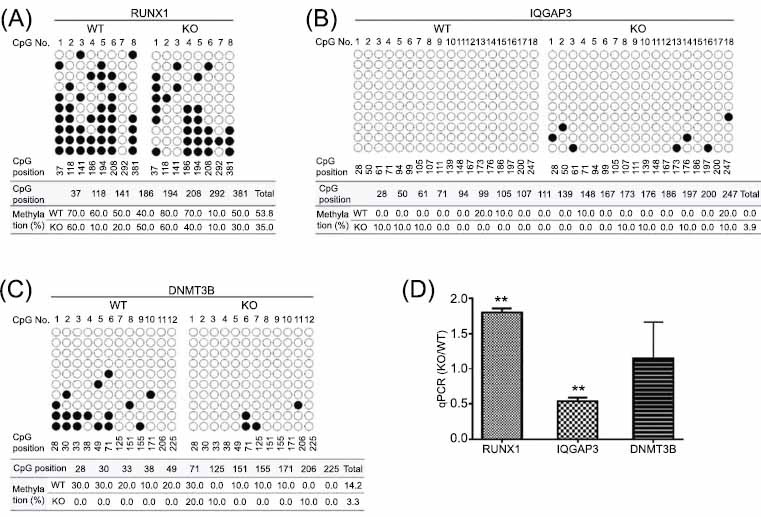
DNA methylation levels and quantitative PCR evaluation of the three representative genes, *RUNX1*, *IQGAP3*, and *DNMT3B*, affected by DNMT3A deficiency. Bisulfite analysis showed that, in comparison with WT cells, the promoter methylation level of *RUNX1* was decreased by DNMT3A deficiency (**A**), while the *IGAPQ3* promoter had more methylated CpG islands in *DNMT3A* KO cells (**B**). *DNMT3B* promoter methylation was also regulated by DNMT3A and decreased upon its knockout (**C**). Quantitative PCR was performed in triplicate for mRNA expression profiles (**D**). In comparison with the relative transcription levels (compared to the *GAPDH* transcription level) in WT cells, *RUNX1* transcription levels were significantly up-regulated by 80%, *IQGAP3* transcription levels were reduced by 46%, and *DNMT3B* transcription levels were up-regulated by about 15%. **p < 0.01 by two-tailed Students’ t-test.

The promoter methylation levels and mRNA transcription levels of *DNMT3B* and two representative tumorigenesis-related genes, *RUNX1* and *IQGAP3*, were verified. The CpG island-rich promoter region (from -1.0 to 0 kb, relative to the transcription start site) was analyzed by BSP for the three genes. According to the results shown in Fig. (**6**), DNMT3A deficiency reduced the DNA methylation level of the *RUNX1* promoter (Fig. **6A**); by contrast, it induced the methylation of the *IQGAP3* promoter (Fig. **6B**). This induction of *IQGAP3* promoter methylation was possibly caused by *DNMT3B*, which should have higher expression level due to the reduced DNA methylation in its promoter region (Fig. **6C**). Quantitative PCR results confirmed the methylation regulation results. The transcription of *RUNX1* was up-regulated by 80%, and that of *IQGAP3* was reduced by 46%. The transcription of *DNMT3B* was also elevated in *DNMT3A* KO cells but only by 15% (Fig. **6D**). This is the first study to show that DNMT3A contributes to the methylation of *DNMT3B*, indicating the cross-activity of the two d*e novo* DNA methyltransferases.

## 4 DISCUSSION

In recent years, DNMT3A has been intensely studied for its role in tumor prognosis and therapy [23, 24]. To better reveal the functions of DNMT3A in cancer occurrence and development, in this study, we mutated HEK293T cells using the CRISPR/Cas9 technology and successfully created a *DNMT3A* knockout cell line with homozygous frameshift deletion in both alleles. LC-MS analysis showed that knockout of *DNMT3A* induced a 21.5% reduction of global DNA methylation. The reduction in DNA methylation could be attributed to the functions of DNMT1 and DNMT3B [11]. In addition, we attempted to mutate *DNMT1* and *DNMT3B* using the same strategy in HEK293T cells, but single stable clones with the required gene mutations or deficiencies were not obtained (data not shown).

**Fig. (7):**
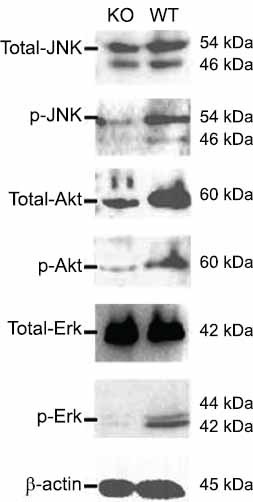
DNMT3A deficiency suppressed MAPK and PI3K-Akt pathways. Total proteins, as well as phosphorylated fractions of Erk, JNK, and Akt, were detected using β-actin as the housekeeping control.

Several studies have focused on *DNMT3A* gene knockout in human or mouse-sourced cells, including human embryonic stem cells, human leukemia cells K562, mouse hematopoietic stem cells, and mouse somatic cells [2, 11, 25, 26]. Compared to mouse cells, human cells are less tolerant to DNMT3A deficiency, which can cause lethality and genomic instability in the cells. The results of these previous studies were consistent with our observations that DNMT3A deficiency suppresses HEK293T cell activity. The doubling time of cells dropped from 0.99 ± 0.28 days to 1.53 ± 0.39 days; this result was similar to the phenomenon of impaired cell growth caused by the *DNMT3A* mutation in K562 cells [2]. We assume that this effect is associated with the MAPK and PI3K-Akt pathways, which predominantly contribute to cell proliferation and migration. In fact, we did observe inhibition of the Erk, JNK, and Akt signaling pathways (Fig. **7**). These changes can be mediated by protein-protein interactions. For instance, altered methylation patterns may lead to changes in the expression of Ras proteins, which are upstream activators of the MAPK pathway. Reduced Ras activity can impede signaling through the MEK/ERK cascade, leading to decreased cellular proliferation [27]. Moreover, DNMT3A deficiency may lead to changes in the methylation status of the PTEN gene, resulting in its upregulation. PTEN is a tumor suppressor that negatively regulates the PI3K pathway, and its increased expression can lead to decreased Akt activation, further impairing cell growth and survival signaling [28].

A previous study introduced frameshift mutations at exons 2 and 3 of *DNMT3A*, ablating it from more upstream regulatory regions [2]. However, in our study, the *DNMT3A* mutation was targeted at exon 19, which encodes the catalytic domain. The reduced genome-wide DNA methylation level in *DNMT3A* KO cells was expected to result in higher transcription levels. However, we unexpectedly observed that a high number of genes were down-regulated in our significant differential expression spectrum, with FDR *p*-values ≤ 0.05 and fold changes ≥ 2 (binomial *p*-value = 7.6×10^-3^). We also found that the top ten genes in the most significant gene cluster were down-regulated.

DNMT3B showed abnormal up-regulation upon DNMT3A deficiency (Fig. **6**) and possibly had a methylation function for some of the genes. Notably, the relationship between DNMT3A deficiency and DNMT3B gene expression and methylation levels is intricate, involving multiple regulatory layers and feedback mechanisms that shape the genomic methylation landscape. In the absence of DNMT3A, the methylation status of the DNMT3B promoter may change, leading to its hypomethylation and subsequent increased expression, as observed in Fig. (**6**). This upregulation of DNMT3B is thought to be a compensatory mechanism to sustain some level of methylation activity in response to DNMT3A deficiency. Additionally, various transcription factors and epigenetic regulators may influence DNMT3B expression. For instance, transcription factors that are typically silenced by DNMT3A could become activated due to altered methylation patterns, impacting DNMT3B expression [29]. Furthermore, the activation of pathways, such as PI3K-Akt or MAPK, may upregulate transcription factors that promote DNMT3B expression. In summary, DNMT3A deficiency affects DNMT3B expression and methylation status through mechanisms, such as altered promoter methylation, feedback regulation, and the influence of other regulatory factors. Understanding these interactions is vital for elucidating the roles of DNA methyltransferases in health and disease, particularly in cancer contexts where mutations in these enzymes are common.

However, new research indicates that two SU(VAR)3-9 homologs, the transcriptional anti-silencing factor SUVH1 and SUVH3, as methyl reader candidates, are associated with euchromatic methylation *in vivo* [30]. In plant, yeast, and mammalian cells, ectopic recruitment of DNAJ1 was shown to enhance gene transcription [31]. Therefore, the SUVH proteins bind to methylated DNA and recruit DNAJ proteins to enhance proximal gene expression, counteracting the repressive effects of transposon insertion near genes [32]. The top ten differently expressed genes were likely associated with the SUVH1 and SUVH3 factors when methylation was decreased. However, the real reason for the down-regulation of the top ten genes in this study is still unknown.

Further investigation of the regulated pathways helped us understand that the lower growth rate is a consequence of DNMT3A deficiency. The calcium signaling pathway and ECM-receptor interaction, which are genetically associated with the progression and recurrence of atrial fibrillation [32], and the Hippo signaling pathway were up-regulated in *DNMT3A* KO cells (FDR *p* values were 0.002449, 0.004114, and 0.03080, respectively). Twist2 is known to regulate ITGA6 and CD44 expression in the ECM-receptor interaction pathway to promote kidney cancer cell proliferation and invasion [33]. The major functions of the Hippo pathway are the restriction of tissue growth in adults and the modulation of cell proliferation, differentiation, and migration in developing organs [34]. Cysteine and methionine metabolism are strictly indispensable to the proliferation of porcine adipogenic precursor cells. Moreover, Met deficiency in media has also been shown to affect the differentiation into adipocytes and alter lipid accumulation [35]. However, due to the complexity of epigenetic modifications, the mechanism of DNMT3A functions remains to be further elucidated.

## CONCLUSION

DNMT3A has been identified to be an ideal target for the development of personalized treatment or prediction of tumor prognosis [23]. Our work is the first report on the effect of the disruption of catalytic domain of DNMT3A on genomic DNA methylation and expression. The genes revealed by RNA-seq to be tightly regulated by DNMT3A in HEK293T cells in this study are of great significance to understanding the functions of DNMT3A in the origin and development of tumors and are potential novel targets for future cancer therapy.

## AUTHORS’ CONTRIBUTIONS

The authors confirm their contribution to the paper as follows: study conception and design: M.Z, J.W, H.L,; Methadology: G.Q, H.Y, N.Z; Data collection: L.X, J.Z; Data Analysis or Interpretation: Q.T; Investigation: L.F; Data Curation: X.P, J.Z. All authors reviewed the results and approved the final version of the manuscript.

## Figures and Tables

**Fig. (1):**
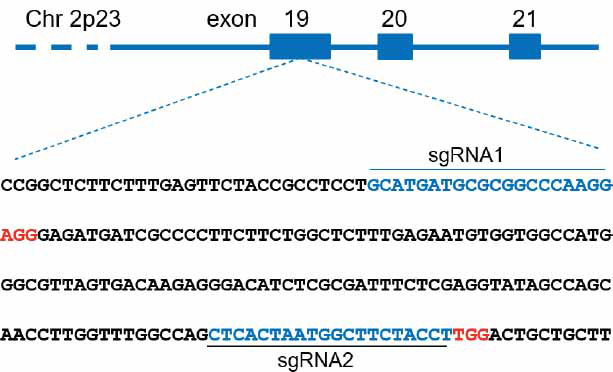
Location and design of sgRNAs. The sgRNA sequences are shown in blue, and protospacer adjacent motif (PAM) bases are shown in red.

**Fig. (3):**
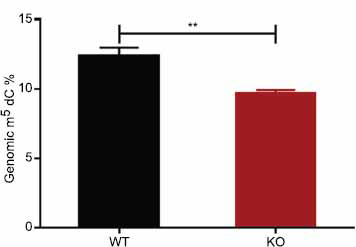
Comparison of methylation levels of genomic DNA between wild-type (WT) and *DNMT3A* knockout (KO) HEK293T cells. The methylation level of genomic DNA decreased by 21.5% due to DNMT3A deficiency. **p < 0.01 by two-tailed Students’ t-test.

**Fig. (4):**
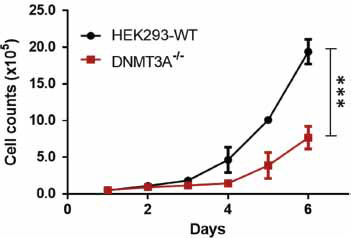
DNMT3A deficiency impaired cell growth. WT: wild-type cells; KO: *DNMT3A* KO cells. Both curves show the mean ± SD (n=6). ***p < 0.001 indicates a significant difference as determined by an unpaired t-test.

**Table 1:** Highly differentially expressed genes.

**Gene ID**	**Gene Name**	**FC**	**log2FC**	***p*-value**	**p-adjust**	**Regulate**
ENSG00000128591	FLNC	0.0363	-4.78	2.17E-175	4.00E-171	down
ENSG00000161671	EMC10	0.009	-6.79	4.81E-164	4.44E-160	down
ENSG00000019549	SNAI2	0.0632	-3.98	1.35E-97	8.32E-94	down
ENSG00000165512	ZNF22	0.0252	-5.31	1.58E-95	7.31E-92	down
ENSG00000184368	MAP7D2	0.0452	-4.47	1.99E-80	7.35E-77	down
ENSG00000173530	TNFRSF10D	0.033	-4.92	2.00E-72	6.15E-69	down
ENSG00000164853	UNCX	0.0516	-4.27	9.81E-70	2.58E-66	down
ENSG00000121413	ZSCAN18	0.0452	-4.47	3.45E-51	7.95E-48	down
ENSG00000148798	INA	0.0868	-3.53	1.22E-48	2.51E-45	down
ENSG00000131435	PDLIM4	0.0543	-4.20	1.34E-45	2.46E-42	down

**Note:** Gene ID: Ensembl IDs. FC: fold change between the two samples. Log2FC: the base is the base 2 logarithm of the difference between the two samples; wild type is the control. *p*-value: The difference in test results for the gene between the two samples. p-adjust: Checked result for *p*-value. Regulate: To indicate whether the expression of the gene is down-regulated or up-regulated; “down” indicates down-regulation of gene expression.

**Table 2:** Enrichment analysis of up-regulated pathways.

**Database**	**ID**	**Term**	***p*-value**	**p-adjusted**
KEGG Pathway	hsa05416	Viral myocarditis	9.17E-06	0.002449
KEGG Pathway	hsa04020	Calcium signaling pathway	9.92E-06	0.002449
KEGG Pathway	hsa04512	ECM-receptor interaction	3.29E-05	0.004115
KEGG Pathway	hsa04142	Lysosome	8.43E-05	0.006504
KEGG Pathway	hsa04614	Renin-angiotensin system	0.000512	0.01489
KEGG Pathway	hsa05410	Hypertrophic cardiomyopathy	0.000845	0.01988
KEGG Pathway	hsa04974	Protein digestion and absorption	0.001509	0.030804
KEGG Pathway	hsa04392	Hippo signaling pathway -multiple species	0.001559	0.030804
KEGG Pathway	hsa04360	Axon guidance	0.002453	0.04179
KEGG Pathway	hsa05231	Choline metabolism in cancer	0.002919	0.048069

**Note:** Term: KEGG pathway name. Database: the KEGG database contains two sub-libraries; one is the KEGG pathway, and the other is the KEGG disease. ID: KEGG pathway ID. The uncorrected *p*-value is provided in the column with the heading ‘*p*-value’; the smaller the *p*-value, the more statistically significant the difference, and a *p*-value of less than 0.05 is considered significant enrichment. p-adjusted: The corrected *p*-value.

**Table 3:** Enrichment analysis of down-regulated pathways.

**Database**	**ID**	**Term**	***p*-value**	**p-adjusted**
KEGG Pathway	hsa03008	Ribosome biogenesis in eukaryotes	9.9E-07	0.0005
KEGG Pathway	hsa00270	Cysteine and methionine metabolism	7.4E-05	0.0103
KEGG Pathway	hsa05205	Proteoglycans in cancer	0.00097	0.0854
KEGG Pathway	hsa00670	One carbon pool by folate	0.00104	0.0854
KEGG Pathway	hsa00230	Purine metabolism	0.00258	0.1621
KEGG Pathway	hsa05169	Epstein-Barr virus infection	0.0027	0.1621
KEGG Pathway	hsa03010	Ribosome	0.00313	0.1621

**Note:** Term: KEGG pathway name. Database: the KEGG database contains two sub-libraries; one is the KEGG pathway, and the other is KEGG disease. ID: KEGG pathway ID. The uncorrected *p*-value is provided in the column with the heading ‘*p*-value’; the smaller the *p*-value, the more statistically significant the difference, and a *p*-value of less than 0.05 indicates significant enrichment. p-adjusted: The corrected *p*-value.

## Data Availability

RNA-seq data are available in the open-access Genome Sequence Archive (gsa.big.ac.cn) under accession no. CRA002294.

## LIST OF ABBREVIATIONS

AML1: Acute Myeloid Leukemia 1
CBFA2: Core-binding Factor Subunit alpha-1
DNMT: DNA Methyltransferase
IQGAP3: IQ Motif Containing GTPase Activating Protein 3
KEGG: Kyoto Encyclopedia of Genes and Genomes
RUNX1: Runt-related Transcription Factor 1

## SUPPLEMENTARY MATERIAL

Supplementary material is available on the publisher’s website along with the published article. The following supporting information can be downloaded at: www.mdpi.com/xxx/s1, Fig. **S1**: Promoter region sequences and CpG islands of representative genes for bisulfite DNA analysis.; Table **S1**: Standard curves of dG and m5dC for the determination of genomic DNA methylation using UPLC-MS.

## References

[1] You J.S., Jones P.A. (2012). Cancer genetics and epigenetics: Two sides of the same coin?. Cancer Cell.

[2] Banaszak L.G., Giudice V., Zhao X., Wu Z., Gao S., Hosokawa K., Keyvanfar K., Townsley D.M., Gutierrez-Rodrigues F., Fernandez Ibanez M.P., Kajigaya S., Young N.S. (2018). Abnormal RNA splicing and genomic instability after induction of DNMT3A mutations by CRISPR/Cas9 gene editing.. Blood Cells Mol. Dis..

[3] Chen F., Zhang Y., Shen L., Creighton C.J. (2024). The DNA methylome of pediatric brain tumors appears shaped by structural variation and predicts survival.. Nat. Commun..

[4] Zhong F., Lin Y., Zhao L., Yang C., Ye Y., Shen Z. (2023). Reshaping the tumour immune microenvironment in solid tumours *via* tumour cell and immune cell DNA methylation: From mechanisms to therapeutics.. Br. J. Cancer.

[5] Bestor T.H. (1992). Activation of mammalian DNA methyltransferase by cleavage of a Zn binding regulatory domain.. EMBO J..

[6] Laranjeira A.B.A., Hollingshead M.G., Nguyen D., Kinders R.J., Doroshow J.H., Yang S.X. (2023). DNA damage, demethylation and anticancer activity of DNA methyltransferase (DNMT) inhibitors.. Sci. Rep..

[7] Okano M., Bell D.W., Haber D.A., Li E. (1999). DNA methyltransferases Dnmt3a and Dnmt3b are essential for de novo methylation and mammalian development.. Cell.

[8] Okano M., Xie S., Li E. (1998). Cloning and characterization of a family of novel mammalian DNA (cytosine-5) methyltransferases.. Nat. Genet..

[9] Huang Y.H., Su J., Lei Y., Brunetti L., Gundry M.C., Zhang X., Jeong M., Li W., Goodell M.A. (2017). DNA epigenome editing using CRISPR-Cas SunTag-directed DNMT3A.. Genome Biol..

[10] Riggs A.D., Xiong Z. (2004). Methylation and epigenetic fidelity.. Proc. Natl. Acad. Sci. USA.

[11] Liao J., Karnik R., Gu H., Ziller M.J., Clement K., Tsankov A.M., Akopian V., Gifford C.A., Donaghey J., Galonska C., Pop R., Reyon D., Tsai S.Q., Mallard W., Joung J.K., Rinn J.L., Gnirke A., Meissner A. (2015). Targeted disruption of DNMT1, DNMT3A and DNMT3B in human embryonic stem cells.. Nat. Genet..

[12] Challen G.A., Sun D., Jeong M., Luo M., Jelinek J., Berg J.S., Bock C., Vasanthakumar A., Gu H., Xi Y., Liang S., Lu Y., Darlington G.J., Meissner A., Issa J.P.J., Godley L.A., Li W., Goodell M.A. (2012). Dnmt3a is essential for hematopoietic stem cell differentiation.. Nat. Genet..

[13] Ghoshal B., Picard C.L., Vong B., Feng S., Jacobsen S.E. (2021). CRISPR-based targeting of DNA methylation in *Arabidopsis thaliana* by a bacterial CG-specific DNA methyltransferase.. Proc. Natl. Acad. Sci. USA.

[14] Villiger L., Joung J., Koblan L., Weissman J., Abudayyeh O.O., Gootenberg J.S. (2024). CRISPR technologies for genome, epigenome and transcriptome editing.. Nat. Rev. Mol. Cell Biol..

[15] Cong L., Ran F.A., Cox D., Lin S., Barretto R., Habib N., Hsu P.D., Wu X., Jiang W., Marraffini L.A., Zhang F. (2013). Multiplex genome engineering using CRISPR/Cas systems.. Science.

[16] Mali P., Yang L., Esvelt K.M., Aach J., Guell M., DiCarlo J.E., Norville J.E., Church G.M. (2013). RNA-guided human genome engineering *via* Cas9.. Science.

[17] Zheng Q., Cai X., Tan M.H., Schaffert S., Arnold C.P., Gong X., Chen C.Z., Huang S. (2014). Precise gene deletion and replacement using the CRISPR/Cas9 system in human cells.. Biotechniques.

[18] Dwi Putra S.E., Neuber C., Reichetzeder C., Hocher B., Kleuser B. (2014). Analysis of genomic DNA methylation levels in human placenta using liquid chromatography-electrospray ionization tandem mass spectrometry.. Cell. Physiol. Biochem..

[19] Trapnell C., Pachter L., Salzberg S.L. (2009). TopHat: Discovering splice junctions with RNA-Seq.. Bioinformatics.

[20] Trapnell C., Roberts A., Goff L., Pertea G., Kim D., Kelley D.R., Pimentel H., Salzberg S.L., Rinn J.L., Pachter L. (2012). Differential gene and transcript expression analysis of RNA-seq experiments with TopHat and Cufflinks.. Nat. Protoc..

[21] Stengel A., Kern W., Meggendorfer M., Nadarajah N., Perglerovà K., Haferlach T., Haferlach C. (2018). Number of RUNX1 mutations, wild-type allele loss and additional mutations impact on prognosis in adult RUNX1-mutated AML.. Leukemia.

[22] Lin M., Liu Y., Ding X., Ke Q., Shi J., Ma Z., Gu H., Wang H., Zhang C., Yang C., Fang Z., Zhou L., Ye M. (2019). E2F1 transactivates IQGAP3 and promotes proliferation of hepatocellular carcinoma cells through IQGAP3-mediated PKC-alpha activation.. Am. J. Cancer Res..

[23] Gao X.N., Yan F., Lin J., Gao L., Lu X.L., Wei S.C., Shen N., Pang J.X., Ning Q.Y., Komeno Y., Deng A.L., Xu Y.H., Shi J.L., Li Y.H., Zhang D.E., Nervi C., Liu S.J., Yu L. (2015). AML1/ETO cooperates with HIF1α to promote leukemogenesis through DNMT3a transactivation.. Leukemia.

[24] Yang S.M., Huang C.Y., Shiue H.S., Pu Y.S., Hsieh Y.H., Chen W.J., Lin Y.C., Hsueh Y.M. (2016). Combined effects of DNA methyltransferase 1 and 3A polymorphisms and urinary total arsenic levels on the risk for clear cell renal cell carcinoma.. Toxicol. Appl. Pharmacol..

[25] Jeong M., Park H.J., Celik H., Ostrander E.L., Reyes J.M., Guzman A., Rodriguez B., Lei Y., Lee Y., Ding L., Guryanova O.A., Li W., Goodell M.A., Challen G.A. (2018). Loss of Dnmt3a immortalizes hematopoietic stem cells *in vivo*.. Cell Rep..

[26] Hatazawa Y., Ono Y., Hirose Y., Kanai S., Fujii N.L., Machida S., Nishino I., Shimizu T., Okano M., Kamei Y., Ogawa Y. (2018). Reduced Dnmt3a increases Gdf5 expression with suppressed satellite cell differentiation and impaired skeletal muscle regeneration.. FASEB J..

[27] Ritt D.A., Abreu-Blanco M.T., Bindu L., Durrant D.E., Zhou M., Specht S.I., Stephen A.G., Holderfield M., Morrison D.K. (2016). Inhibition of Ras/Raf/MEK/ERK pathway signaling by a stress-induced phospho-regulatory circuit.. Mol. Cell.

[28] Hu T., Chen F., Chen D., Liang H. (2022). DNMT3a negatively regulates PTEN to activate the PI3K/AKT pathway to aggravate renal fibrosis.. Cell. Signal..

[29] Loaeza-Loaeza J., Beltran A.S., Hernández-Sotelo D. (2020). DNMTs and impact of CpG content, transcription factors, consensus motifs, lncRNAs, and histone marks on DNA methylation.. Genes (Basel).

[30] Du J., Johnson L.M., Groth M., Feng S., Hale C.J., Li S., Vashisht A.A., Gallego-Bartolome J., Wohlschlegel J.A., Patel D.J., Jacobsen S.E. (2014). Mechanism of DNA methylation-directed histone methylation by KRYPTONITE.. Mol. Cell.

[31] Harris C.J., Scheibe M., Wongpalee S.P., Liu W., Cornett E.M., Vaughan R.M., Li X., Chen W., Xue Y., Zhong Z., Yen L., Barshop W.D., Rayatpisheh S., Gallego-Bartolome J., Groth M., Wang Z., Wohlschlegel J.A., Du J., Rothbart S.B., Butter F., Jacobsen S.E. (2018). A DNA methylation reader complex that enhances gene transcription.. Science.

[32] Büttner P., Ueberham L., Shoemaker M.B., Roden D.M., Dinov B., Hindricks G., Bollmann A., Husser D. (2018). Identification of central regulators of calcium signaling and ECM–receptor interaction genetically associated with the progression and recurrence of atrial fibrillation.. Front. Genet..

[33] Zhang H.J., Tao J., Sheng L., Hu X., Rong R.M., Xu M., Zhu T.Y. (2016). Twist2 promotes kidney cancer cell proliferation and invasion by regulating ITGA6 and CD44 expression in the ECM-receptor interaction pathway.. OncoTargets Ther..

[34] Meng Z., Moroishi T., Guan K.L. (2016). Mechanisms of Hippo pathway regulation.. Genes Dev..

[35] Castellano R., Perruchot M.H., Tesseraud S., Métayer-Coustard S., Baeza E., Mercier Y., Gondret F. (2017). Methionine and cysteine deficiencies altered proliferation rate and time-course differentiation of porcine preadipose cells.. Amino Acids.

