# Supplementary material for: DNMT3A Deficiency Reduces DNMT3B Gene Methylation and Contributes to Whole-genome Transcription Alterations in HEK293 Cells
Source: Curr Genomics. 2025 Feb 24;26(5):389–99. doi: 10.2174/0113892029351729250217113313 (PMC12728590; doi:10.2174/0113892029351729250217113313)
Supplement: Supplementary file 1 [file CG-26-5-389_SD1.pdf]

Supplementary Material

DNMT3A Deficiency Reduces *DNMT3B* Gene Methylation and Contributes to Whole-genome Transcription Alterations in HEK293 Cells

Mengxiao Zhang[1,#], Jiaxian Wang[1,2,#], Gen Qi[1], Lanfeng Xie[3], Qiuxiang Tian[4,5], Hui Yang[1], Lei Feng[6], Nan Zhu[7], Xingchen Pan[5], Jianwei Zhu[1], Jianjun Hu[3,*], Peng Chen[4,5,*] and Huili Lu[1,*]

[1]Engineering Research Center of Cell and Therapeutic Antibody, Ministry of Education, School of Pharmacy, Shanghai Jiao Tong University, Shanghai, China; [2]Department of Hematology, VU University Medical Center, Amsterdam, Netherlands; [3]Department of Infectious Disease, Tongren Hospital, School of Medicine, Shanghai Jiao Tong University, Shanghai, China; [4]Key Laboratory of Pathobiology, Ministry of Education, Jilin University, Changchun, Jilin, China; [5]Department of Genetics, College of Basic Medical Sciences, Jilin University, Changchun, Jilin, China; [6]Instrumental Analysis Center, Shanghai Jiao Tong University, Shanghai, China; [7]Shanghai General Hospital, School of Medicine, Shanghai Jiao Tong University, Shanghai, China

Table S1. Promoter region sequences and CpG islands of representative genes for bisulfite DNA analysis.

No	Gene	CpG island Sequences	Product Length	CpGs
1	RUNX 1	tttcccaggctttaaaatacctgtgagttgccagcccgttgtaggggtcagactctcac-caaaacatttctttttatttatttttccctttttatagtttcacttatgccatgatagacgt-taccaaggacttaactctcccggagctgatgcctagcattttaaatgatgg-gatccacatcctgtcggagcagcggcttgatgccagcgttgaattactattgaataagcag-caatgaaatctttatcaaaataatcagtagttccaaaaaccacaaataacaacag-gagccgagttgtactaaatcagcaaagaccattgagatataataagtgactgagtcac-ttttttctacatcccccccctcttgcaaagtctactcggctattttcttgcacagcctgggggagggcaggt	416 bp	8
2	IQGAP 3	cagaaaaggagcctggaaggaacaagacgaggaactgctgtaaggggagcgg-tactgccccggcctggggcgcctcttctcccagcacctgggcggcccggctccgcggccgg-gaactacaaatcccaggattctcagcggtgtggacgggaagtgtcctgtctggcggtgccgacggtgaggggcggtggcccaacggcgggagat-gaagtgtcctgtctggcggtgccgacggtgaggggcggtggcccaacggcgggagat-tcaaacctggaagaaggaggaacatggagaggagagcagcgggcccaggctgggcagcctgtgagtg	274 bp	18
3	DNMT 3B	ccaaagcaggatgacaggcaggggcaccgcggcgccccggtggcactgcggctg-gaggtgggggttaaagcggagactctggtgctgtgtgactacag-tgggggccctgccctctctgagcccccgcctccaggcctgtgtgtgtgtctccgttcgggttgaaaggagcccgggaaaaaggccccagaaggagtc tggttttggacgtctgaccccacccctcccgcttagggcttctgatcccccaggg	250 bp	12

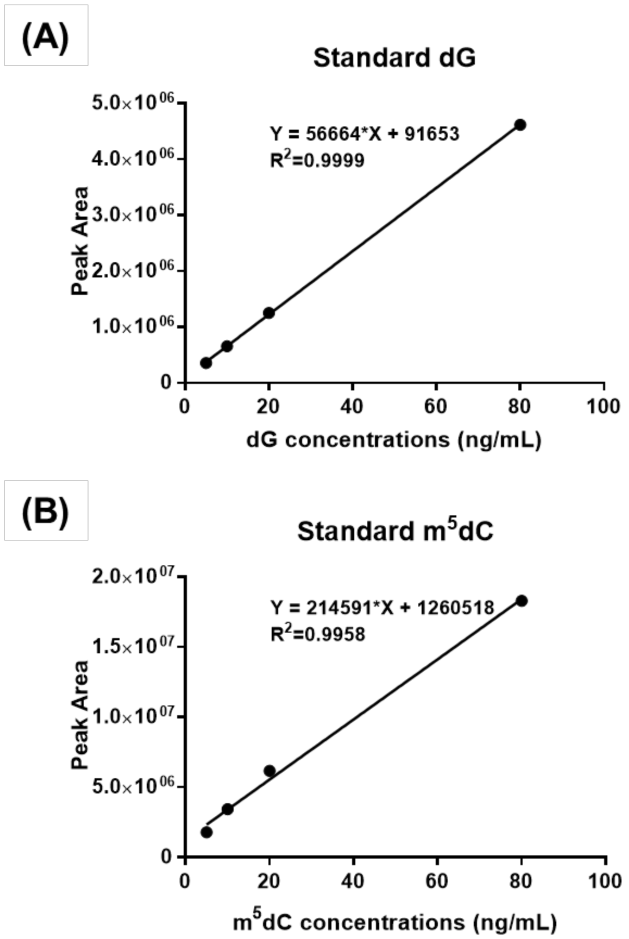

Fig. (S1). Standard curves of dG and m5dC for determination of genomic DNA methylation using UPLC-MS.
